# The memory clinic and psychosocial intervention: Translating past promise into current practices

**DOI:** 10.3389/fresc.2023.1052244

**Published:** 2023-05-04

**Authors:** Esme Moniz-Cook, Gail Mountain

**Affiliations:** ^1^ Faculty of Health Sciences, University of Hull, Hull, United Kingdom; ^2^ Centre for Applied Dementia Studies, University of Bradford, Bradford, United Kingdom

**Keywords:** dementia, proactive, memory clinic, stigma, primary care, care-coordination

## Abstract

Disproportionate negative effects since the pandemic have amplified the already limited post-diagnostic support for older people with dementia. This paper summarizes an exploratory randomized controlled study of a proactive family-based intervention compared with “usual” post-diagnostic dementia care. Memory clinic practitioners collaborated with the family doctor (GP) to coordinate this. At 12-month follow-up, positive effects on mood, behavior, carer coping and maintenance of care at home were found. Current approaches to deliver post-diagnostic support in primary care may require rethinking since (i) GP workloads have increased with low numbers of GPs per head of population in parts of England; and (ii) unlike many other long-term conditions, ongoing stigma, fear and uncertainty associated with dementia adds to the huge complexity of timely care provision. There is a case for return to a “one-stop facility”, with a single pathway of continuing multidisciplinary coordinated care for older people with dementia and families. Future longitudinal research could compare structured post-diagnostic psychosocial intervention coordinated by skilled practitioners in a single locality memory service “hub”, against other approaches such support organized mostly within primary care. Dementia-specific instruments for outcome measurement are available for use in routine practice, and should be included in such comparative studies.

## Introduction

The past three decades has seen a growth of memory clinics for the diagnosis and treatment of dementia worldwide, with many services also acting as vehicles of best practice, innovation and research (1). As far back as 1997, the scope for psychosocial research in memory clinics was outlined (2). In the UK memory clinics hosted studies of reminiscence therapy, cognitive rehabilitation, cognitive stimulation, occupational therapy and interventions to maintain independence and wellbeing in people with dementia (3–8). England's 2009 National Dementia Strategy (NDS) (9) and governmental calls for memory clinics to be available in “every town and city” (10) raised hopes for counteracting the known fear of a dementia-diagnosis and stigma associated with mental health services for older people (11–13).

Box 1 **Looking ahead: Memory clinics and post-diagnostic dementia care.**

• An exploratory memory clinic RCT of a proactive family-based intervention demonstrated positive outcomes on the person's mood, reported behaviour problems, carer burden and maintenance of care at home at 12-month follow-up.
• Key to timely intervention appears to be skilled ongoing care-coordination incorporated in the multidisciplinary team and shared tasks with GPs.
• Family-based therapeutic support for managing uncertainty, fear and stigma during diagnosis and through the “journey with dementia” is a potentially important mechanism of change in application of psychosocial intervention.
• Given current demands on GPs in England and the influence of fear, uncertainty, stigma and social withdrawal in older people with dementia, a properly resourced “one stop collaborative facility” for older people with dementia and families is recommended.
• Future longitudinal studies could compare care-coordination within this collaborative approach against other approaches to post-diagnostic care.
• Valid instruments for evaluating effectiveness should be used to examine which service model has best mid-long term outcomes for people with dementia and families.

Interest in the scope for family doctors (GPs) to provide post-diagnostic psychosocial support emerged (14), as did primary care dementia collaborative innovation worldwide (15). Various protocols each with their own aims appeared in the literature. Examples included geriatricians or psychiatrists augmenting work in primary care, dementia practitioners supported by psychiatry or geriatricians working in primary care and integrated working between primary care and old age psychiatry (16–21). However, early on, health policy initiatives in England did not convince some GPs to take on the responsibility for managing dementia (22). The situation does not appear to have changed. The PriDem study across England and Wales notes that despite some financial incentives, GPs may not have the capacity to deliver good quality dementia care (23). European primary care studies also report problems in GP engagement in delivery of dementia initiatives (24, 25). Reasons for fragile post-diagnostic dementia care include fragmentation, poor communication around care pathways and inadequate health and social care policies (26, 27).

The introduction of memory clinics (1, 2), a later focus on primary care (14), the development of practice guidance (28, 29) and psychosocial intervention research (3–8), does not seem to have translated into delivery of timely psychosocial intervention. A large cohort study noted limited support (30), a significant care gap was found when families in distress were referred to specialists (31) and people and their families continue to experience many barriers to receiving post-diagnostic support (32).

Timely psychosocial intervention requires good knowledge of family function and associated psychosocial need(s) (33–35). Many factors influence decisions to access support with differing expectations and ways of living with dementia (36–39). Early encounters during the uncertain transition to dementia (40) requires skilled family “psycho-diagnostics” and communication (33–35, 41). Studies conducted during the pandemic confirm previous knowledge about the variation of ongoing psychological need(s) in families. During the restrictions, some appeared resilient, others reported significant negative impacts, some initially resilient family carers became anxious as time went on and as restrictions were eased improvements in family experience varied (42–44). Locality variation in service pathways (26) also occurred. Some services closed whilst others attempted to reconfigure care (45).

Access to post-diagnostic care has been a challenge for people with dementia and families (30–32). This paper considers how we can improve the offer of proactive tailored dementia care for older people. It begins by describing and exploratory memory clinic collaborative care RCT, from more than two decades ago. It considers strengths, limitations relevant to practice, and potential mechanisms of change underlying proactive intervention in dementia. Looking ahead to today's context, we outline a rationale for re-thinking the organization of post-diagnostic dementia care, with suggestions for policy related research.

### An exploratory ‘memory clinic-primary care liaison’ RCT

This RCT built on an earlier innovation to examine the effects of proactive individualised intervention for people with dementia and families (46, 47). Described as “primary care liaison”, the multidisciplinary memory clinic (i.e., psychiatry, geriatric medicine and clinical psychology) collaborated with GPs to share tasks.

#### Research question

“Can early memory clinic intervention delivered with GPs, reduce “excess disability” (i.e., psychological burden) in people with dementia and families?”

#### Intervention

Both experimental and control families attended for diagnosis with a psychiatrist, clinical psychologist and nurse. This used visual representation of the brain and neuropsychological test results to communicate cognitive strengths, reasons for everyday difficulties and helpful strategies (48). People could ask questions and received a personalized advice booklet summarising the meeting. Control families then received “usual care” from community dementia teams consisting of old age psychiatry, nurses and social work. Experimental families received care from the memory clinic care-coordinator (a nurse or graduate psychologist) supervised by a clinical psychologist. The care-coordinator delivered post-diagnostic support and communicated with the GP and memory clinic specialists. The intervention described elsewhere (46, 48) involved clinic or home visits to check on family understanding of information and agree relevant interventions. Choices included health considerations including drug review or monitoring for timely treatment of health conditions such as infection (organised by GPs) or access to a geriatrician or old age psychiatrist (organised by the care co-ordinator). Psychosocial intervention considered training in external memory aides (anxiety prevention) and social-behavioural activation (depression prevention) (46, 48). Communication skills training from a clinical psychologist was available, using video-assisted materials for managing ongoing challenges such as repeated questioning and agitated disorientation.

#### Study procedures and participants

Following memory clinic assessment, families consented to randomisation (conducted by an independent service) to the experimental or “usual care” support. Stratification occurred for cognition (MMSE >24) but not for a “significant other” since families were located for all participants. Measures of cognition, mood, reported behaviour, carer coping, burden, the person's psychotropic medication usage, number of intervention contacts and home-care maintenance, occurred at baseline, 6 and 12 months (Table 1). Over six months 48 people met the inclusion criteria of diagnosis of dementia—DSM-IV criteria, ≥65 years, mild-moderate dementia—MMSE score ≥14 (Supplementary Figure S1).

**Table 1:** Demographics, mood, behaviour, carer coping/burden, maintenance of care at home, use of psychotropic medication and intervention contacts.

	Experimental Group (*n* = 25)			Control Group (*n* = 23)			
Patient Age—years—mean (sd)	77.6 (4.54)			75.9 (5.00)			
Carer's Age—years—mean (sd)	72.6 (9.05)			67.4 (14.58)			
Diagnosis AD—Alzheimer's Disease *n* (%)	13 (52)			13 (57)			
VAD—Vascular Dementia *n* (%)	12 (48)			10 (43)			
**MEASURES mean—**$\bar{x}$ **(sd)**	**Months**			**Months**			***P* value**
** **	0 *n* = 25	6 *n* = 24	12 *n* = 23	0 *n* = 23	6 *n* = 19	12 *n* = 15	Estimate (95% CI)
Cognition: MMSE^a^	23.08 (4.34)	22.42 (5.51)	22.38 (4.43)	21.7 (3.53)	21.75 (5.66)	20.8 (5.39)	0.059
Mood: Depression Cornell Scale^b^	2.84 (2.69)	3.04 (2.93)	2.38 (3.49)	5.3 (4.09)	6.65 (5.00)	7.23 (5.33)	0.008;3.15 (.883,5.41)
Mood: Anxiety: HAD-A Scale^c^	5.24 (3.96)	4.25 (3.27)	4.91 (3.52)	6.26 (3.48)	6.42 (3.91)	6.13 (2.72)	N.S.
Reported Memory and Behaviour Problems^d^	18.05 (8.97)	17.79 (9.55)	10.81 (7.91)	22.17 (13.14)	27.15 (16.11)	20.0 (13.11)	0.005;7.31 (1.96,12.7)
Reported Non-Cognitive Symptoms^e^	0.71 (0.69)	1.13 (1.74)	0.65 (0.99)	1.07 (1.02)	0.75 (1.02)	1.43 (1.02)	0.025^†^
Carer Management of Memory and Behaviour Problems^d^	14.56 (13.27)	10.08 (7.52)	6.81 (6.69)	20.35 (16.54)	19.1 (16.31)	13.54 (7.59)	0.012;5.21 (1.01,9.41)
Carer Management of Mood^d^	6.0 (8.61)	3.46 (4.85)	1.90 (2.59)	6.52 (7.29)	7.1 (7.62)	5.31 (4.87)	0.010;3.83 (.953,6.70)
Carer Day-to-day Concerns^f^	13.60 (9.24)	13.67 (11.96)	9.95 (9.9)	19.96 (13.58)	18.75 (16.07)	18.77 (13.61)	0.032
Carer Competence^g^	28.59 (6.74)	28.68 (4.68)	30.9 (4.23)	26.89 (5.68)	26.26 (5.28)	27.0 (5.99)	0.035;−3.21 (−6.19,.239)
Carer Anxiety: HAD-A Scale^c^	3.50 (3.69)	4.57 (4.02)	4.38 (4.22)	6.18 (3.54)	6.14 (4.45)	5.13 (5.21)	N.S.
Carer Depression: HAD: HAD-D Scale^c^	2.63 (2.37)	2.78 (2.78)	2.71 (3.12)	3.18 (2.28)	4.38 (3.54)	4.00 (3.34)	N.S.
Carer Psychological Health: GHQ^h^	2.42 (2.90)	3.04 (4.03)	2.77 (5.36)	5.29 (6.02)	4.19 (6.13)	4.27 (7.11)	N.S.
**OTHER MEASURES** (*n* = total sample)	*n* = 25	*n* = 25	*n* = 25	*n* = 23	*n* = 23	*n* = 23	
Breakdown of care at home (number) (includes deaths and admissions to care)	0	0	2	0	2	8	0.022^††^
Use of psychotropic medication Patient Number (%) any psychotropic	9 (36%)	8 (32%)	8 (32%)	8 (35%)	10 (43%)	11 (48%)	N.S.
Total psychotropic drugs used*	11	10	10	8	13	14	
Number of intervention contacts $\bar{x}$ (sd) median	0	5.28 (3.08) 5	2.60 (3.14) 2	0	3.39 (4.38) 2	3.10 (4.76) 1	0.06^†††^

^a^ Mf F. Folstein SE. McHugh PR.“Mini-mental state.” A practical method for grading the cognitive state of patients for the clinician. J Psychiatr Res. 1975;12(3):189–98. Abrams RC, Young RC, Shamoian CA. Cornell scale for depression in dementia. Biological

^b^ Alexopoulos GS, Abrams RC, Young RC, Shamoian CA. Cornell scale for depression in dementia. Biological Psychiatry. 1988 Feb 1;23(3):271–84.

^c^ Zigmond AS, Snaith RP. The hospital anxiety and depression scale. Acta psychiatrica scandinavica. 1983 Jun; 67(6):361–70.

^d^ Teri L, Truax *P*, Logsdon R, Uomoto J, Zarit S, Vitaliano PP. Assessment of behavioral problems in dementia: the revised memory and behavior problems checklist. Psychology and aging. 1992 Dec;7(4):622.

^e^ Allen NH, Gordon S, Hope T, Burns A. Manchester and Oxford Universities scale for the psychopathological assessment of dementia (MOUSEPAD). The British Journal of Psychiatry. 1996 Sep;169(3):293–307.

^f^ Gilleard CJ. Living with dementia: Community care of the elderly mentally infirm. Routledge; 1984.

^g^ Vernooy-Dassen MJ, Felling AJ, Brummelkamp EP, Dauzenberg MG, van den Bos GA, Grol RP. Assessment of caregiver's competence in dealing with the burden of caregiving for a dementia patient: a short sence of competence questionnaire (SSCQ) suitable for clinical practice. Journal of the American Geriatrics Society. 1999;47:256–7.

^h^ Goldberg DP. Manual of the general health questionnaire (GHQ-28). NFER-Nelson: Windsor, UK. 1981.

* Some patients were on more than 1 psychotropic drug at one time.

^†^ Scores on this measure were not normally distributed. Mann-Whitney test used on reported symptoms at 12 months minus reported symptoms at 6 months.

^††^ *p* value obtained from Pearson's chi squared test for an association between intervention group and break down of care occurring by 12 months.

^†††^ *p* value obtained from a Mann-Whitney test for a difference between intervention groups in the total number of contacts in 12 months.

**Analysis**“Quality of life (QoL)” outcome measures (except Non-Cognitive Symptoms) at 6 and 12 months were analysed using a mixed-model ANOVA with random patient effects, adjusting for corresponding baseline measures, diagnosis and age of patient. For such measures for which significant group differences but not group × time interactions were found, *P*-values, group difference estimates and 95% confidence intervals for the underlying group difference (control—experimental) are presented. For other measures, the comparisons to which the *P*-values apply are in the main text. “Use of psychotropic medication”: percentages are percentages of those who started in the group. All available data was used so that numbers analysed varied from measure to measure. Modelling used R Version 1.3.1

#### Summary of results

Table 1 summarizes the findings of this RCT. Cognition deteriorated in both groups (*F *= 3.815, df = 1.36, *P *= 0.0586 for the main effect of time), but the experimental intervention had a better impact on mood (depression: *F *= 7.870, df = 1.42, *P *= 0.0076 for the main effect of group) and reported memory and behaviour problems (*F* = 8.883, df = 1.42, *P *= 0.0048 for the main effect of group). Also better in the intervention group were: carer coping with their relative's mood (*F *= 7.213, df = 1.43, *P *= 0.0102 for the main effect of group) and memory and behaviour problems (*F *= 6.84, df = 1.41, *P *= 0.0124 for the main effect of group); and their sense of competence (*F *= 4.809, df = 1.35, *P *= 0.0350 for the main effect of group). The between group difference in number of intervention contacts was not marked (*U *= 144.5, *P *= 0.06), but for the experimental group there were significantly more family contacts in the first six months (Mann-Whitney *U *= 164.5, *P *= 0.011), but not in the second six months (Mann-Whitney *U *= 192.5, *P *= 0.501). The group by time interactions for reported non-cognitive symptoms for patients (Mann-Whitney *U *= 72, *P *= 0.025) and carer day-to-day concerns (*F *= 5.033, df = 1.32, *P *= 0.0319) was seen at 12 months follow-up. Maintenance of care at home was also significantly better in the experimental group (92% E vs. 65% C; *P *= 0.022). The control group increased their proportion of psychotropic drug usage (35%–48%), whilst this decreased slightly (36%–32%) in the experimental group (see Table 1).

#### Commentary

There are few RCTs of proactive multi-component psychosocial intervention for older people with dementia and families. This exploratory RCT was conducted in one locality (population 274,000) prior to availability of AchEIs for Alzheimer's disease. As such, it was a *defacto* study of care practice. This study demonstrates that proactive intervention can minimise depression and reported behavioural problems in people with dementia, and improve family coping. Tailored intervention offered shortly after the diagnostic meeting, showed positive effects 12 months later.

#### Practice strengths

This shared-care collaboration between GPs and the memory clinic continued in this locality for 12 years beyond the RCT. It evolved to include other geriatricians, neurology, social workers and a pharmacist. More GPs adopted the shared-care arrangements with practice nurses or receptionists coordination between them and the memory clinic. Memory clinic care-coordinators extended to more graduate psychologists, nurses, occupational therapists and social work, each supporting a minimum of 50 families annually. They had daily access to the multidisciplinary team for advice and treatment. Some coordinators supervised new support staff, thus doubling the annual number of families supported. Family diagnostics (34, 35) facilitated case management protocols to avoid overlooking people without a “significant other” or families at risk of distress.

#### Limitations

Similar to other countries (20, 24, 25), communication could be weak since not all GPs fully adopted the collaborative approach. Consequentially, when changed behaviour associated with potential new health conditions occurred, care-coordinators could struggle to engage the GP. Many families did not have the awareness or confidence to access timely medical help but this hurdle was managed through family education on how to gain access to GP review of potential ill health.

Loss of research staff disallowed planned longitudinal data beyond 12 months. Treatment data for ongoing conditions such as infection was collected (47) but analysis was not completed. A more recent longitudinal study in another region of England shows that continuity of GP care for people with dementia ≥65 years has safer prescribing and lower rates of major adverse events (49).

Primary vs. secondary outcomes were not specified (47), as one aim was to explore the potential of instruments for responsiveness to psychosocial intervention. Analysis was limited to known instruments of psychological burden such as measures of mood, behaviour and carer coping.

Finally, audit of content of psychosocial intervention for all 48 participants (47) was not analysed. Neither was in-depth process evaluations or intervention costs considered.

### Potential mechanisms of change

Despite aforementioned limitations, we can use contemporary studies to consider how the memory clinic might have worked to deliver the outcomes.

(i) The negative social effects of a dementia-diagnosis, the continued role of stigma and the view that “nothing can be done” (50, 51) remain concerning. Although we did not measure stigma, some components can be conceived as “stigma-neutralising”(48). For example during diagnosis, use of cognitive assessments to explain assets and reasons for reported concerns to “separate brain from mind” (48) resonates with a recent qualitative study of neuropsychological-informed communication during diagnostic disclosure (52).
(ii) All families received the diagnostic procedure with personalised written information, but this was not enough. Early on, experimental families received what is now seen as important psychological support (53), with the potential spin-off of creating a psychosocial environment of “safe uncertainty” (48, 54). This may have mitigated against fear and stigma associated with diagnosis (11, 50, 51), thus empowering families to activate other intervention components (55) and may explain the positive effects on mood and family coping.
(iii) Memory clinic personalised support ranged from face to face meetings, telephone “checks” and increased effort to engage friends in accompanying people to available activities, when families were unavailable to assist. Studies using manualised psychosocial interventions delivered by trained practitioners are now available to offer this type of support (5–8)
(iv) Clarity of shared tasks is key to delivery of timely care (15, 26). Memory clinic practitioners addressed psychosocial need(s), whilst GPs and primary care practitioners covered health need(s). Care-coordinators facilitated each service to alert the other when needs occurred. They communicated with the GP service or accessed others such as psychiatrists, geriatricians, pharmacists and social workers where relevant. This continuity of care may have contributed to outcomes at 12 months, such as the need for psychotropic medication.

## Discussion

Dementia is a complex condition, predominately affecting older people. Continuity of timely care requires both specialised psychosocial family-based support as well as attention to ongoing multi-morbidity and polypharmacy. Decentralising memory clinics and transferring activity to primary care is thought to facilitate continuity of care (26). However, this may not be always sustainable within England's NHS. GP workloads have increased with low numbers of GPs per head of population in some localities. Whist GP continuity can have good medical outcomes (49) decision-making for complex multi-morbidity in ageing populations is not easy, particularly where communication gaps persist between them and specialist physicians (56, 57). Additionally, weak organisational systems can undermine collaboration (58) and case management in primary care has huge challenges (59).

There are pragmatic advantages of clearly defined case management arrangements with memory clinic practitioners coordinating personalized family interventions, collaborating with GPs or specialist geriatricians for the management of medical conditions.

This creates space for skilled support to:

(i) balance tensions of failed expectations early on during diagnostic disclosure (37);
(ii) facilitate positive coping strategies such as addressing ongoing effects of stigma-related social withdrawal (50);
(iii) arrange ongoing access to psychosocial expertise in tailoring support, given the complexity associated with wide-ranging ways in which people and families perceive and manage dementia (38, 39);
(iv) offer ready access to psychological expertise to discuss often avoided ongoing difficult conversations (60);
(v) coach (55) families in recognition of potential comorbid health conditions and accessing relevant health care (61).

Memory clinic-based continuing case management has scope to improve the quality of post-diagnostic care and avoid families being left with limited support and “nowhere to turn to” (30, 32).

### Looking ahead: proactive psychosocial intervention and memory clinics

This RCT used proactive multicomponent psychosocial interventions with a 12-month follow-up. It mitigated low mood and reported behaviour problems as well as facilitating carer coping and maintenance of care at home. A nurturing relationship from a skilled care-coordinator and the family at the start of diagnosis was key to stigma-neutralising support and continuing care. This subtle skilled family-based work during and following diagnosis, allowed dementia-specific professionals to learn together to engage with the various ways in which families function (34, 35), and review ongoing support that families needed. As such, the memory clinic itself became a psychosocial intervention.

The goals of proposed intervention are also important. This RCT intervention focussed on family-based support to reduce “excess disabilities” associated with non-cognitive aspects of dementia such as reduced mood, behaviour changes and carer burden. There is now a literature of newer studies aimed at supporting people to “live well” with the condition through participating in pleasurable activities and social networks (3–8).

The PRIDEM research programme studied six case sites, all with primary care links. Of these, three were “secondary care-led” of which one had a drop-in facility. The authors note that no one approach was perfect and all sites delivered some aspects of good quality care (26, 62). They distinguish between roles of a “named point of contact” and a “care-coordinator” (see Bamford et al. p13 table 7 (62)). The pan-European ActifCare study highlights the need to integrate roles of the “named point of contact” and the case manager (63). Our RCT intervention, incorporated care-coordination (i.e., case management) as the named practitioner contact from the start, where the arrangement continued with the family through their journey with dementia.

Primary care could manage the task of post-diagnostic dementia care with additional financial resources for quality, over and above the “quantity-measured” reimbursements that are currently available (64). This may work where particular GP surgeries have strong leaders and dementia-specific nurses (26). However, most UK GP surgeries unlike hospitals are *defacto* small businesses and not all might accept reimbursement for this complex condition that involves an ongoing battle against stigma, fear and uncertainty for many families (11, 50, 51, 53).

Revisiting a proactive memory service “one stop shop” (65) with skilled family-based work to meet the varied and changeable needs of older people with dementia and families is a practical alternative. A skilled proactive family-based “coaching” (55) approach underlies timely dementia care. It contrasts with the “point of contact” notion, where the responsibility is on people with dementia to decide if they need support. It additionally avoids the risks of pathway-fragmentation when psychosocial specialists are required (31).

Overall, the pandemic has disproportionately negatively affected the care and quality of life of people with dementia and families (66, 67). There is an urgent case for properly resourcing a “single point” (65) post-diagnostic community memory service for older people with dementia and their families, particularly where the numbers of GPs per head of population is low.

Going forward, we recommend research focussed on a collaborative transdisciplinary (15) community memory clinic-led configuration i.e., a “one stop hub” (65) with integrated dementia-skilled coordination for the continuing care of older people with dementia. This could involve a locality-based non-linear interactive dementia network to include GP leaders and other primary care opportunities (68). Evaluation should use valid instruments (69, 70) to measure outcomes for individuals and families (see Box 1).

## Conclusion: time for change

Decades on, it is, we suggest *time for change* in the organization of post-diagnostic dementia care. We present a case for properly resourcing memory clinics to spearhead proactive timely psychosocial intervention and related research. Lessons from the past highlight the advantages of organizing locality-based memory services with resources to sustain opportunities for older people and their families, through: (i) establishing a trusting relationship with a skilled care-coordinator, supported by multi-professional dementia expertise from the start; (ii) using proactive structured ongoing personalized planning; and (iii) paying assertive attention to inequalities (63).

Overall, the literature is thin on comparative studies of organizational models for delivering psychosocial interventions to minimise disabilities and maintain quality of life in older people with dementia. Future longitudinal research could use validated instruments (69, 70) to compare conceptually driven post-diagnostic psychosocial intervention coordinated by skilled practitioners in a single locality memory service collaborative “hub” (68), against other post-diagnostic care models (26).

## Data Availability

The raw data supporting the conclusions of this article will be made available by the authors, without undue reservation.

## In Memoriam

Peter Campion (Emeritus Professor of Primary Care Medicine, University of Hull; PhD (sociology, Liverpool) 1996, BM, BCh (Oxford) was a co-applicant and drafted “Participants and results”; in the month before his premature death due to progressive supranuclear palsy (December 2021), he made suggestions on the commentary and discussion.

## References

[1] Jolley D Moniz-Cook E. Memory clinics in context. Indian J Psychiatry. (2009) 51(Suppl 1):S70–6. PMID: ; PMCID: 21416022 PMC3038532

[2] Moniz-Cook E Woods RT. The role of memory clinics and psychosocial intervention in the early stages of dementia. Int J Geriatr Psychiatry. (1997) 12(12):1143–5. 10.1002/(SICI)1099-1166(199712)12:12<1143::AID-GPS707>3.0.CO;2-F 9444536

[3] Woods RT Orrell M Bruce E Edwards RT Hoare Z Hounsome B REMCARE: pragmatic multi-centre randomised trial of reminiscence groups for people with dementia and their family carers: effectiveness and economic analysis. PloS One. (2016) 11(4):e0152843. 10.1371/journal.pone.0152843 27093052 PMC4836678

[4] Orrell M Yates L Leung P Kang S Hoare Z Whitaker C The impact of individual Cognitive Stimulation Therapy (iCST) on cognition, quality of life, caregiver health, and family relationships in dementia: a randomised controlled trial. PLoS Med. (2017) 14(3):e1002269. 10.1371/journal.pmed.1002269 28350796 PMC5369684

[5] Clare L Kudlicka A Oyebode JR Jones RW Bayer A Leroi I Individual goal-oriented cognitive rehabilitation to improve everyday functioning for people with early-stage dementia: a multicentre randomised controlled trial (the GREAT trial). Int J Geriatr Psychiatry. (2019) 34(5):709–21. 10.1002/gps.5076 30724405 PMC6593854

[6] Wenborn J O’Keeffe AG Mountain G Moniz-Cook E King M Omar RZ Community occupational therapy for people with dementia and family carers (COTiD-UK) versus treatment as usual [Valuing active life in dementia (VALID)] study: a single-blind, randomised controlled trial. PLoS Med. (2021) 18(1):e1003433. 10.1371/journal.pmed.1003433 33395437 PMC7781374

[7] Csipke E Moniz-Cook E Leung P Yates L Birt L Walton H Feasibility and acceptability evaluation of the promoting independence in dementia (PRIDE) intervention for living well with dementia. Int Psychogeriatr. (2021) 33(6):601–14. 10.1017/S1041610220001386 32847643

[8] Mountain GA Cooper CL Wright J Walters SJ Lee E Craig C The journeying through dementia psychosocial intervention versus usual care study: a single-blind, parallel group, phase 3 trial. Lancet Healthy Longev. (2022) 3(4):e276–85. 10.1016/S2666-7568(22)00059-9 36098301

[9] Department of Health. Living well with dementia: A national dementia strategy. London: Department of Health (2009).

[10] Eaton L. Every town and city in England to have a memory clinic, says health secretary. Br Med J. 338:b464. 10.1136/bmj.b464 19193687

[11] Moniz-Cook E Manthorpe J Carr I Gibson G Vernooij-Dassen M. Facing the future: a qualitative study of older people referred to a memory clinic prior to assessment and diagnosis. Dementia. (2006) 5(3):375–95. 10.1177/1471301206067113

[12] Jolley D Moniz-Cook E. Memory clinics are all about stigma, not screening. Br Med J. (2009) 338:b860. 10.1136/bmj.b860 19258365

[13] Mukadam N Livingston G. Reducing the stigma associated with dementia: approaches and goals. Aging Health. (2012) 8(4):377–86. 10.2217/ahe.12.42

[14] Robinson L Iliffe S Brayne C Goodman C Rait G Manthorpe J DeNDRoN primary care clinical studies group primary care and dementia: 2. Long-term care at home: psychosocial interventions, information provision, carer support and case management. Int J Geriatr Psychiatry. (2010) 25(7):657–4. 10.1002/gps.2405 19946862

[15] Galvin JE Valois L Zweig Y. Collaborative transdisciplinary team approach for dementia care. Neurodegener Dis Manag. (2014) 4(6):455–69. 10.2217/nmt.14.47 25531688 PMC4308691

[16] Christley J Cuenca J Davis KJ Evry N Hartwell T Shepherd K. Evaluation of the geriatrician in the practice model of care for dementia assessment and management in rural Australia. Aust J Rural Health. (2022) 30(1):55–64. 10.1111/ajr.12824 35064952

[17] Greaves I Greaves N Walker E Greening L Benbow SM Jolley D. Gnosall primary care memory clinic: eldercare facilitator role description and development. Dementia. (2015) 14(4):389–408. 10.1177/1471301213497737 24339104

[18] Austrom M G Damush TM West Hartwell C Perkins T Unverzagt F Boustani M Development and implementation of nonpharmacologic protocols for the management of patients with Alzheimer's Disease and their families in a multiracial primary care setting. Gerontologist. (2004) 44(4):548–53. 10.1093/geront/44.4.548 15331812

[19] Lai SH Tsoi T Tang CT Hui RJ Tan KK Yeo YW An integrated, collaborative healthcare model for the early diagnosis and management of dementia: preliminary audit results from the first transdisciplinary service integrating family medicine and geriatric psychiatry services to the heart of patients’ homes. BMC Psychiatry. (2019) 19(1):1–9. 10.1186/s12888-018-1996-0 30736756 PMC6368696

[20] Lee L Hillier LM Stolee P Heckman G Gagnon M McAiney CA Enhancing dementia care: a primary care–based memory clinic. J Am Geriatr Soc. (2010) 58(11):2197–204. 10.1111/j.1532-5415.2010.03130.x 20977435

[21] Michalowsky B Xie F Eichler T Hertel J Kaczynski A Kilimann I Cost-effectiveness of a collaborative dementia care management—results of a cluster-randomized controlled trial. Alzheimer's Dementia. (2019) 15(10):1296–308. 10.1016/j.jalz.2019.05.008 31409541

[22] Woods RT Moniz-Cook E Iliffe S Campion P Vernooij-Dassen M Zanetti O Dementia: issues in early recognition and intervention in primary care. J R Soc Med. (2003) 96(7):320–4. 10.1177/014107680309600703 12835442 PMC539533

[23] Wheatley A Brunskill G Dow J Bamford C Poole M Robinson L The primary care annual dementia review: a qualitative study of the views and experiences of service users and providers. *medRxiv*. (2022). 10.1101/2022.04.26.22274255.

[24] Seidel K Quasdorf T Haberstroh J Thyrian JR. Adapting a dementia care management intervention for regional implementation: a theory-based participatory barrier analysis. Int J Environ Res Public Health. (2022) 19(9):5478. 10.3390/ijerph19095478 35564877 PMC9101206

[25] Balsinha C Iliffe S Dias S Freitas A Barreiros FF Gonçalves-Pereira M. Dementia and primary care teams: obstacles to the implementation of Portugal's Dementia strategy. Prim Health Care Res Dev. (2022) 23:1–8. 10.1017/S1463423621000876 PMC8919178 35177149

[26] Robinson L. An evidence-informed, primary care-based, task-shared approach to post-diagnostic dementia care: the PriDem programme In World Alzheimer Report 2022: Life After Diagnosis: Navigating Treatment, Care and Support (2022). Alzheimer's Disease International. p. 140–141.

[27] Martin A O’Connor S Jackson C. A scoping review of gaps and priorities in dementia care in Europe. Dementia. (2020) 19(7):2135–51. 10.1177/1471301218816250 30497303 PMC7917562

[28] https://www.dementiavoices.org.uk/wp-content/uploads/2021/02/a_guide_to_psychosocial_interventions_in_dementia.pdf.

[29] Patel B Perera M Pendleton J Richman A Majumdar B. Psychosocial interventions for dementia: from evidence to practice. Adv Psychiatr Treat. (2014) 20(5):340–9. 10.1192/apt.bp.113.011957

[30] van Horik JO Collins R Martyr A Henderson C Jones RW Knapp M Limited receipt of support services among people with mild-to-moderate dementia: findings from the IDEAL cohort. Int J Geriatr Psychiatry. (2022) 37(3):1–8. 10.1002/gps.5688 PMC9306706 35128725

[31] Manthorpe J Hart C Watts S Goudie F Charlesworth G Fossey J Practitioners’ understanding of barriers to accessing specialist support by family carers of people with dementia in distress. Int J Care Caring. (2018) 2(1):109–23. 10.1332/239788218X15187915193354

[32] Giebel C Robertson S Beaulen A Zwakhalen S Allen D Verbeek H. “Nobody seems to know where to even turn to”: barriers in accessing and utilising dementia care services in England and The Netherlands. Int J Environ Res Public Health. (2021) 18(22):12233.ople. 10.3390/ijerph182212233 34831989 PMC8622725

[33] Gonçalves-Pereira M. Toward a family-sensitive practice in dementia. In: Verdelho A, Gonçalves-Pereira M, editors. Neuropsychiatric symptoms of cognitive impairment and dementia. Switzerland: International Publishing (2017). p. 349–68. 10.1007/978-3-319-39138-0

[34] Moniz-Cook ED Laidlaw K Knight B. Assessment and psychosocial intervention for older people with suspected dementia: a memory clinic perspective. In: Laidlaw K Knight B, editors. Handbook of emotional disorders in late life: Assessment and treatment. Oxford & New York: Oxford University Press (2008). p. 421–51.

[35] Moniz-Cook E Rewston C. Choosing psychosocial interventions for people with dementia and their families. In: Manthorpe J Moniz-Cook E, editors. Timely psychosocial interventions in dementia care: Evidence-based practice London & Philadelphia: Jessica Kingsley Publishers (2020). p. 30–47.

[36] Linden I Hevink M Wolfs C Perry M Dirksen C Ponds R. Understanding patients’ and significant others’ preferences on starting a diagnostic trajectory for dementia: an integrative review. Aging Ment Health. (2022):1–2. 10.1080/13607863.2022.2084505. [Epub ahead of print] 35763442 PMC10166060

[37] Karnieli-Miller O Werner P Aharon-Peretz J Sinoff G Eidelman S. Expectations, experiences, and tensions in the memory clinic: the process of diagnosis disclosure of dementia within a triad. Int Psychogeriatr. (2012) 24(11):1756–70. 10.1017/S1041610212000841 22687191

[38] Clare L Gamble LD Martyr A Quinn C Litherland R Morris RG Psychological processes in adapting to dementia: illness representations among the IDEAL cohort. Psychol Aging. (2022) 37(4):524–41. 10.1037/pag0000650 34881948 PMC9134708

[39] Bjørkløf GH Helvik AS Ibsen TL Telenius EW Grov EK Eriksen S. Balancing the struggle to live with dementia: a systematic meta-synthesis of coping. BMC Geriatr. (2019) 19(1):1–24. 10.1186/s12877-019-1306-9 31666020 PMC6822397

[40] Campbell S Manthorpe J Samsi K Abley C Robinson L Watts S Living with uncertainty: mapping the transition from pre-diagnosis to a diagnosis of dementia. J Aging Stud. (2016) 37:40–7. 10.1016/j.jaging.2016.03.001 27131277

[41] Dahm MR Crock C. Understanding and communicating uncertainty in achieving diagnostic excellence. JAMA. (2022) 327(12):1127–8. 10.1001/jama.2022.2141 35238876

[42] O'Rourke G Pentecost C Van Den Heuvel E Victor C Quinn C Hillman A Living with dementia under COVID-19 restrictions: coping and support needs among people with dementia and carers from the IDEAL cohort. Ageing Soc. (2021):1–23. 10.1017/S0144686X21001719. [Epub ahead of print]

[43] Daley S Farina N Hughes L Armsby E Akarsu N Pooley J COVID-19 and the quality of life of people with dementia and their carers—the TFD-C19 study. PloS One. (2022) 17(1):e0262475. 10.1371/journal.pone.0262475 35045120 PMC8769363

[44] Altieri M Santangelo G. The psychological impact of COVID-19 pandemic and lockdown on caregivers of people with dementia. Am J Geriatr Psychiatry. (2021) 29(1):27–34. 10.1016/j.jagp.2020.10.009 33153872 PMC7577876

[45] Wheatley A Poole M Robinson L. Changes to post-diagnostic dementia support in England and Wales during the COVID-19 pandemic: a qualitative study. BMJ Open. (2022) 12(2):e059437. 10.1136/bmjopen-2021-059437 35110326 PMC8811272

[46] Moniz-Cook E Agar S Gibson G Win T Wang M. A preliminary study of the effects of early intervention with people with dementia and their families in a memory clinic. Aging Ment Health. (1998) 2(3):199–211. 10.1080/13607869856687

[47] Moniz-Cook E Campion P. Early Psychosocial Intervention through a memory clinic: A Randomised Controlled Trial protocol registration. Website (2019). Available at: https://www.cochranelibrary.com/central/doi/10.1002/central/CN-01797634/full (Accessed July 7, 2022).

[48] Moniz-Cook E Gibson G Harrison J Wilkinson H. Timely psychosocial interventions in a memory clinic. In: Moniz-Cook E Manthorpe J, editors. Early psychosocial interventions in dementia: Evidence-based practice. London: Jessica Kingsley (2008). p. 50–70.

[49] Delgado J Evans PH Gray DP Sidaway-Lee K Allan L Clare L Continuity of GP care for patients with dementia: impact on prescribing and the health of patients. Br J Gen Pract. (2022) 72(715):e91–8. 10.3399/BJGP.2021.0413 35074796 PMC8803082

[50] Amano T Reynolds A Scher C Jia Y. The effect of receiving a diagnosis of Alzheimer's Disease and related dementias on social relationships of older adults. Dement Geriatr Cogn Disord. (2021) 50(4):401–6. 10.1159/000519581 34649243

[51] Gauthier S Rosa-Neto P Morais JA Webster C. World Alzheimer report 2021: Journey through the diagnosis of dementia. Alzheimer's Disease International. London (2021). Available at: https://www.alzint.org/u/World-Alzheimer-Report-2021.pdf accessed 12/07/22

[52] Gruters AA Ramakers IH Stiekema AP Verhey FR Kessels RP de Vugt ME. An exploratory study of the development and pilot testing of an interactive visual tool of neuropsychological test results in memory clinics. J Alzheimers Dis. (2021) 79(3):1157–70. 10.3233/JAD-201128 33386807 PMC7990417

[53] Xanthopoulou P McCabe R. Subjective experiences of cognitive decline and receiving a diagnosis of dementia: qualitative interviews with people recently diagnosed in memory clinics in the UK. BMJ Open. (2019) 9(8):e026071. 10.1136/bmjopen-2018-026071 31375604 PMC6688685

[54] Mason B. Towards positions of safe uncertainty. Human Syst. (2022) 2(2):54–63. 10.1177/26344041211063125

[55] Van’t Leven N De Lange J der Ploeg ES Pot AM. Working mechanisms of dyadic, psychosocial, activating interventions for people with dementia and informal caregivers: a qualitative study. Clin Interv Aging. (2018) 13:1847. 10.2147/CIA.S160363 30310270 PMC6166763

[56] Hughes LD. Understanding the processes behind the decisions–GPs and complex multimorbidity decision making. BMC Primary Care. (2022) 23(1):1. 10.1186/s12875-022-01781-0 PMC9238096 35761167

[57] Timmins L Kern LM O’Malley AS Urato C Ghosh A Rich E. Communication gaps persist between primary care and specialist physicians. Ann Fam Med. (2022) 20(4):343. 10.1370/afm.2781 35879085 PMC9328695

[58] Bunn F Burn AM Robinson L Poole M Rait G Brayne C Healthcare organisation and delivery for people with dementia and comorbidity: a qualitative study exploring the views of patients, carers and professionals. BMJ Open. (2017) 7(1):e013067. 10.1136/bmjopen-2016-013067 28100562 PMC5253574

[59] Iliffe S Waugh A Poole M Bamford C Brittain K Chew-Graham C The effectiveness of collaborative care for people with memory problems in primary care: results of the CAREDEM case management modelling and feasibility study. Health Technol Assess (Rockv). (2014) 18(52):1–48. 10.3310/hta18520 PMC4781405 25138151

[60] Moore KJ Goodison H Sampson EL. The role of the memory service in helping carers to prepare for end of life: a mixed methods study. Int J Geriatr Psychiatry. (2019) 34(2):360–8. 10.1002/gps.5034 30443938 PMC6519218

[61] Häikiö K Sagbakken M Rugkåsa J. Family carers’ involvement strategies in response to sub-optimal health services to older adults living with dementia–a qualitative study. BMC Geriatr. (2020) 20(1):1. 10.1186/s12877-020-01663-z PMC7430106 32807099

[62] Bamford C Wheatley A Brunskill G Booi L Allan L Banerjee S Key components of post-diagnostic support for people with dementia and their carers: a qualitative study. Plos One. (2021) 16(12):e0260506. 10.1371/journal.pone.0260506 34928972 PMC8687564

[63] Stephan A Bieber A Hopper L Joyce R Irving K Zanetti O Barriers and facilitators to the access to and use of formal dementia care: findings of a focus group study with people with dementia, informal carers and health and social care professionals in eight European countries. BMC Geriatr. (2018) 18(1):1–6. 10.1186/s12877-018-0816-1 29866102 PMC5987478

[64] Wheatley A Brunskill G Dow J Bamford C Poole M Robinson L The primary care annual dementia review: a qualitative study of the views and experiences of service users and providers. *medRxiv*. (2022). 2022-04.

[65] Steiner GZ Ee C Dubois S MacMillan F George ES McBride KA “We need a one-stop-shop”: Co-creating the model of care for a multidisciplinary memory clinic with community members, GPs, aged care workers, service providers, and policy-makers. BMC Geriatr. (2020) 20:1–14. 10.1186/s12877-019-1410-x PMC7014614 32046657

[66] Liu KY Howard R Banerjee S Comas-Herrera A Goddard J Knapp M Dementia wellbeing and COVID-19: review and expert consensus on current research and knowledge gaps. Int J Geriatr Psychiatry. (2021) 36(11):1597–639. 10.1002/gps.5567 34043836 PMC8237017

[67] Masterson-Algar P Allen MC Hyde M Keating N Windle G. Exploring the impact of COVID-19 on the care and quality of life of people with dementia and their carers: a scoping review. Dementia. (2022) 21(2):648–76. 10.1177/14713012211053971

[68] Oostra DL Harmsen A Nieuwboer MS Rikkert MG Perry M. Care integration in primary dementia care networks: a longitudinal mixed-methods study. Int J Integr Care. (2021) 21(4):1–12. 10.5334/ijic.5675 PMC8663750 34963758

[69] Moniz-Cook E Vernooij-Dassen M Woods R Verhey F Chattat R Vugt MD A European consensus on outcome measures for psychosocial intervention research in dementia care. Aging Ment Health. (2008) 12(1):14–29. 10.1080/13607860801919850 18297476

[70] Clarke C Woods B Moniz-Cook E Mountain G Øksnebjerg L Chattat R Measuring the well-being of people with dementia: a conceptual scoping review. Health Qual Life Outcomes. (2020) 18:1–4. 10.1186/s12955-020-01440-x 32709238 PMC7382062

