# Supplementary material for: The memory clinic and psychosocial intervention: Translating past promise into current practices
Source: Front Rehabil Sci. 2023 May 4;4:1052244. doi: 10.3389/fresc.2023.1052244 (PMC10192709; doi:10.3389/fresc.2023.1052244)
Supplement: Supplementary file 1 [file Datasheet1.doc]

**Supplementary File 1 Consort figure: Flow of Participants Through Each Stage of the Randomised Controlled Trial (RCT)**

**Referred to Clinic (total sample) n= 149**

Did not attend n= 15

Assessed for eligibility n = 134

**Excluded (n =86)**

Did not meet inclusion criteria for mild dementia i.e. MMSE, >14 (n = 83)

Died (n = 3)

**Randomised (n = 48)**

**Allocated to experimental group (n = 25)**

Received allocated intervention (n = 25 )

Did not receive allocated intervention (n = 0)

**At 6mths: Lost to follow up (n = 1)**

In residential care (n =1)

Discontinued intervention (n = 0)

**At 6mths: Lost to follow up (n = 4)**

In residential care (n = 4)

Discontinued intervention (n = 0)

**At 6mths: Analysed (n = 24)**

**Excluded from analysis (n = 1 )**

In residential care (n =1)

**At 6mths Analysed (n = 19)**

**Excluded from analysis (n = 4)**

In residential care (n = 4)

**Allocated to control group (n = 23)**

Received allocated intervention (n = 23)

Did not receive allocated intervention (n =0)

**At 12mths: Lost to follow up (n = 3)**

In residential care (n =1)

Deceased (n =2)

**At 12mths: Lost to follow up (n = 8)**

In residential care (n = 6)

Deceased (n =2)

**At 12mths: Analysed (n = 22)**

**Excluded from analysis (n = 3)**

In residential care (n =1)

Deceased (n =2)

**At 12mths Analysed (n = 15)**

**Excluded from analysis (n = 8)**

In residential care (n = 6)

Deceased (n = 2)
